# Editorial: The role of inflammation in neurodegenerative diseases

**DOI:** 10.3389/fncel.2023.1192514

**Published:** 2023-04-14

**Authors:** Egor Dzyubenko, Maryam Sardari, Dirk M. Hermann

**Affiliations:** ^1^Department of Neurology and Center for Translational Neuro- and Behavioral Sciences (C-TNBS), University Hospital Essen, Essen, Germany; ^2^Department of Animal Biology, School of Biology, College of Science, University of Tehran, Tehran, Iran

**Keywords:** neuroinflammation, neurodegeneration, microglia, neuroprotection, AIMP1, SIRT2, TSPO, IGF-1R

Neuroinflammation is a critical pathophysiological process implicated in the development of neurodegenerative diseases. It involves the activation of immune cells in the brain, particularly microglia. Targeting chronically activated immune responses and, more specifically, innate immunity has been previously proposed as a promising approach for developing translational therapies in neurodegenerative diseases (Heneka et al., 2014). This Research Topic expands our understanding of the complex molecular mechanisms underlying neuroinflammation and how they contribute to neurodegeneration.

The main innate immune cells of the brain are microglia, the myeloid cells that migrate into nervous tissue during embryonic development (Bennett and Bennett, 2020). Microglia cells are critical regulators of brain homeostasis, which actively patrol the extracellular space (Nimmerjahn et al., 2005) and protect neurons upon brain injury by responding to damage-associated molecular signals (Szalay et al., 2016). In this Research Topic, the original research article by Oh et al. reveals that aminoacyl tRNA synthetase complex-interacting multifunctional protein 1 (AIMP1) is an important mediator of microglial activation during neuroinflammation. The authors found that AIMP1 induced the polarization of microglial cells into the neurodegeneration-associated reactive state, as indicated by the expression of cell surface markers and pro-inflammatory cytokines. Microglial AIMP1 induced the phosphorylation of mitogen-activated protein kinases (MAPKs) and the nuclear factor-kappa B (NF-κB) components, leading to the activation of microglial cells. Inhibition of MAPK and NF-κB pathways suppressed the AIMP1-induced microglial cell activation and neurodegeneration-associated polarization. Pharmacological modulation of neuroinflammatory signaling in microglia is a promising strategy for treating neurodegenerative diseases, Feng et al. show in their study focusing on the translocator protein 18 kDa (TSPO) ligands midazolam and PK11195. The authors investigated the effects of midazolam and PK11195 on the nucleotide-binding domain-like receptor protein 3 (NLRP3) inflammasome signaling and the associated release of inflammatory cytokines in immortalized microglial BV-2 cells. Pretreatment with TSPO ligands significantly reduced the levels of reactive oxygen species (ROS), IL-1β, and IL-18 induced by exposure to lipopolysaccharide (LPS). These results suggest that TSPO ligands could be used to control microglial reactivity in neuroinflammatory diseases. In addition to AIMP1 and TSPO modulation, the selective inhibition of sirtuin 2 (SIRT2) is another promising strategy to treat neuroinflammatory diseases, Fan and Bin highlight in their review within this Research Topic. The authors summarized the key experimental data indicating the pivotal role of SIRT2 in regulating immune responses and its contribution to the pathophysiology of neuroinflammatory conditions including Alzheimer's disease, Parkinson's disease, depression, and stroke. Several SIRT2-selective small-molecule inhibitors are available to date, and the authors discuss their therapeutic potential in neurodegenerative diseases, suggesting that the attenuation of neuroinflammatory responses with SIRT2 inhibitors can promote neuroprotection.

Growing evidence indicates that extracellular vesicles (EVs) mediate intercellular communication in the healthy and diseased brain (Xia et al., 2022). In this Research Topic, Thomas et al. analyzed the role of EVs and the non-coding miRNAs they contain in the pathogenesis of neurodegenerative diseases. The authors discuss the observation that EVs similarly modulate the pathogenesis of glioblastoma and neurodegenerative diseases. They review the key miRNAs associated with both pathologies and suggest miRNA-loaded EVs as novel therapeutic targets. The original research article by Zhuang et al. shows that inhibition of miR-125b promotes neurite outgrowth while suppressing cell apoptosis and proinflammatory cytokines by regulating forkhead box Q1 (FOXQ1), prostaglandin-endoperoxide synthase 2 (PTGS2), and cyclin-dependent kinase 5 (CDK5). FOXQ1 inhibition increased cell apoptosis and proinflammatory cytokines but repressed neurite outgrowth, while PTGS2 inhibition achieved the opposite effects. CDK5 inhibition attenuated cell apoptosis, with less effect on neurite outgrowth and inflammation. Moreover, FOXQ1 inhibition attenuated the effect of miR-125b inhibition on regulating neurite outgrowth, cell apoptosis, and proinflammatory cytokines. Based on their data, the authors conclude that pharmacological inhibition of miR-125b is a promising target for developing novel Alzheimer's disease treatments.

Using a transgenic mouse model of Alzheimer's disease, Sohrabi et al. investigated the impact of insulin-like growth factor-1 receptor (IGF-1R) on amyloid-β (Aβ)-related neurodegeneration. The authors show that IGF-1 deficiency reduces reactive gliosis and Aβ plaque deposition. Moreover, treatment with a selective, competitive, and reversible IGF-1R inhibitor yielded similar protective effects. Based on these results, the authors suggest that small molecule therapy targeting IGF-1R signaling may hold promise for the treatment of Alzheimer's disease.

Evaluating the efficiency of novel therapies requires reliable markers of neuroinflammation and associated neurodegeneration. In this Research Topic, Ruan and Elyaman comment on the possible applications of microglial transmembrane protein 119 (TMEM119) as a marker of activated microglia in neuroinflammatory conditions. The authors provide a critical update about the expression of TMEM119 in the resting and activated microglia and conclude that detecting the extracellular TMEM119 domain in patient's blood or cerebrospinal fluid provides a straightforward method for monitoring the state of microglial activation in neurodegenerative diseases.

In summary, this Research Topic highlights novel molecular mechanisms underlying neuroinflammation and neurodegeneration. New data indicate the critical role of microglia in neuroinflammatory diseases and identify novel therapeutic targets such as AIMP1, TSPO, SIRT2, and miRNA-loaded EVs. This Research Topic also revealed the potential of IGF-1R signaling as a target for Alzheimer's disease treatment. Further investigations are required to evaluate the efficacy of these therapies and identify reliable biomarkers of neuroinflammation in neurodegenerative diseases. The findings reported in this Research Topic provide a significant contribution for the development of clinically applicable therapies targeting innate immune responses in neurodegenerative diseases.

## Author contributions

ED drafted the manuscript. All authors discussed the manuscript and contributed to the final version.

## References

[B1] Bennett M. L. Bennett F. C. (2020). The influence of environment and origin on brain resident macrophages and implications for therapy. Nat. Neurosci. 23, 157–166. 10.1038/s41593-019-0545-6 31792468

[B2] Heneka M. T. Kummer M. P. Latz E. (2014). Innate immune activation in neurodegenerative disease. Nat. Rev. Immunol. 14, 463–477. 10.1038/nri3705 24962261

[B3] Nimmerjahn A. Kirchhoff F. Helmchen F. (2005). Resting microglial cells are highly dynamic surveillants of brain parenchyma *in vivo*. Science 308, 1314–1318. 10.1126/science.1110647 15831717

[B4] Szalay G. Martinecz B. Lenart N. Kornyei Z. Orsolits B. Judak L.. (2016). Microglia protect against brain injury and their selective elimination dysregulates neuronal network activity after stroke. Nat. Commun. 7, 11499. 10.1038/ncomms11499 27139776 PMC4857403

[B5] Xia X. Wang Y. Zheng J. C. (2022). Extracellular vesicles, from the pathogenesis to the therapy of neurodegenerative diseases. Transl. Neurodegener. 11, 53. 10.1186/s40035-022-00330-0 36510311 PMC9743667

